# Transabdominal supra-diaphragmatic self-attached mesh for recurrent type IV diaphragmatic hernia on emergency setting: Case report

**DOI:** 10.1016/j.ijscr.2019.07.036

**Published:** 2019-07-22

**Authors:** Adeodatus Yuda Handaya, Aditya Rifqi Fauzi, Victor Agastya Pramudya Werdana

**Affiliations:** a Digestive Surgery Division, Department of Surgery, Faculty of Medicine, Universitas Gadjah Mada/Dr. Sardjito Hospital, Jl. Kesehatan No. 1, Yogyakarta, 55281, Indonesia; b Faculty of Medicine, Universitas Gadjah Mada/Dr. Sardjito Hospital, Yogyakarta, 55281, Indonesia

**Keywords:** Recurrence, Diaphragmatic hernia, Trans abdominal, Supradiaphragmatic, Self-attached mesh

## Abstract

**Introduction:**

Adult diaphragmatic hernia is a rare case and usually present with various clinical manifestations. Surgery can be done by thoracic and abdominal approaches. In emergency cases, mostly performed by abdominal approach. The use of mesh to close the defect is currently emerging as a viable option.

**Presentation of case:**

We report a case of 70-year-old woman with complaints of shortness of breath and abdominal pain. The patient had a history of laparoscopic surgery with the installation of an anti-adhesive mesh for diaphragmatic hernia seven months before admission. Chest X-ray and Computed Tomography (CT) scan found recurrence of diaphragmatic hernia. The patient then received laparotomy showing diaphragmatic hernia with contents in the form of transverse colon, ileum, gastric and omentum. The self-attached mesh was placed on the supra-diaphragm with abdominal approach. The patient was hospitalised four days after surgery. At two weeks and six months follow-up, no complaints were found. CT scan and chest X-ray were within normal limits.

**Discussion:**

We did a modified technique in managing the recurrence and adhesion of the diaphragmatic hernia using the hernia mesh attached onto the superior side of the diaphragm to minimise the complications and recurrences.

**Conclusions:**

Transabdominal supradiaphragmatic self-attached mesh for recurrent diaphragmatic hernia can be considered to be an alternative treatment for recurrent diaphragmatic hernia in an emergency setting because this procedure is relatively easier, safer, causes fewer postoperative complications, and can prevent recurrence.

## Highlights

• A rare case of adult diaphragmatic hernia.
• Use of mesh to close the defect.
• Transabdominal supradiaphragmatic self-attached mesh placement.
• No complications after surgery.
• Alternative treatment for recurrent diaphragmatic hernia in an emergency setting.

## 1 Introduction

Diaphragmatic hernia is herniation of the intra-abdominal organs through an abnormal defect in the diaphragm. Adult diaphragmatic hernias are a rare condition that occurs mainly due to a direct penetrating injury to the diaphragm (3%–15%) or, less commonly, secondary to blunt abdominal trauma (5–7%) [2,10].

In adults, diaphragmatic hernia is more frequent in males with proportion of 3:1. Most diaphragmatic hernias occur on the left (85%) and without hernia sac (85%). Size of the hernia does not always represent the size of the diaphragmatic defect [[1], [2], [3], [4]].

**Fig. 1:**
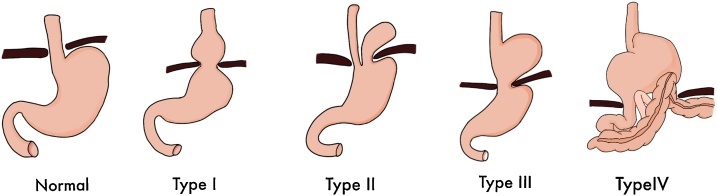
Types of the diaphragmatic hernia.

There are four types of diaphragmatic hernia (Fig. 1). Type I is a sliding hernia, which is the most common diaphragmatic hernia type that occurs and is associated with reflux disease. In type I, phreno-esophageal membrane is widened, so the part of the gastric cardia moves upward. Whereas in type II, the part that is passed through the esophageal hiatus is the gastric fundus. Type III is a combination of types I and II, in which the gastroesophageal junction is also herniated upward but not fixed. The last type is type IV, which involves the herniation of the other abdominal organs, such as ileum, jejunum, colon, pancreas, and other abdominal organs, through the esophageal membrane [11,12].

The thoracic and abdominal approaches are often used as surgery management of diaphragmatic hernia. Currently, there is no guideline for the absolute indication in the timing of the surgery. The use of mesh in the management of diaphragmatic hernia is emerging as a viable option; several studies have shown low recurrence in mesh use. However, there is still a lack of long-term research to prove the safety and effectiveness [13]. This research work has been reported in line with the SCARE checklist [14].

## 2 Presentation of case

**Fig. 2:**
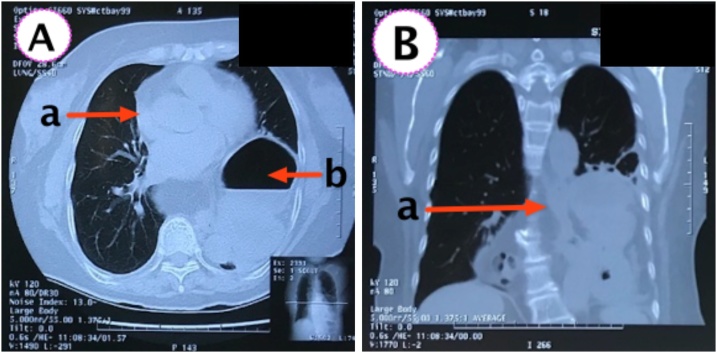
CT scan axial view (A) and Coronal view (B) showed herniation of abdominal visceral to thoracic space (A.a and B.a) and partial gastric outlet obstruction. (A.b).

**Fig. 3:**
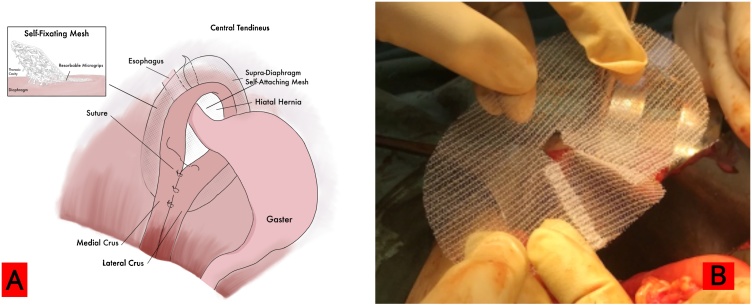
A. Illustration of supradiaphragmatic mesh placement (abdominal view) B. Self-attached mesh.

**Fig. 4:**
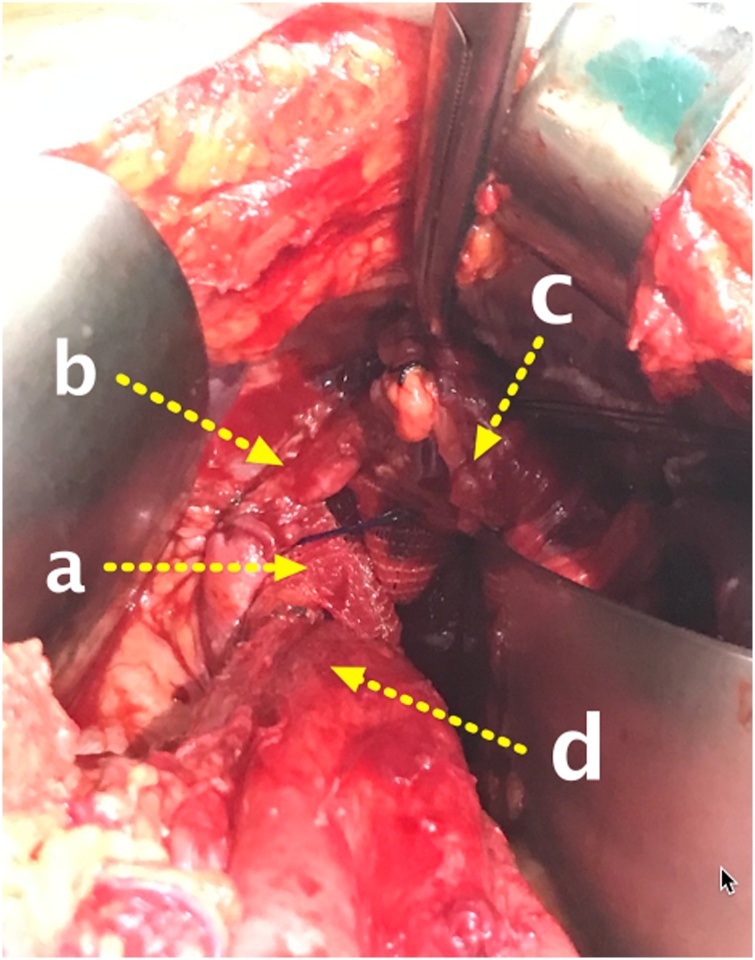
Supradiaphragmatic self-attached mesh placement by abdominap approach. (a) through between medial (b) and lateral (c) crus at gastroesophageal junction (d).

**Fig. 5:**
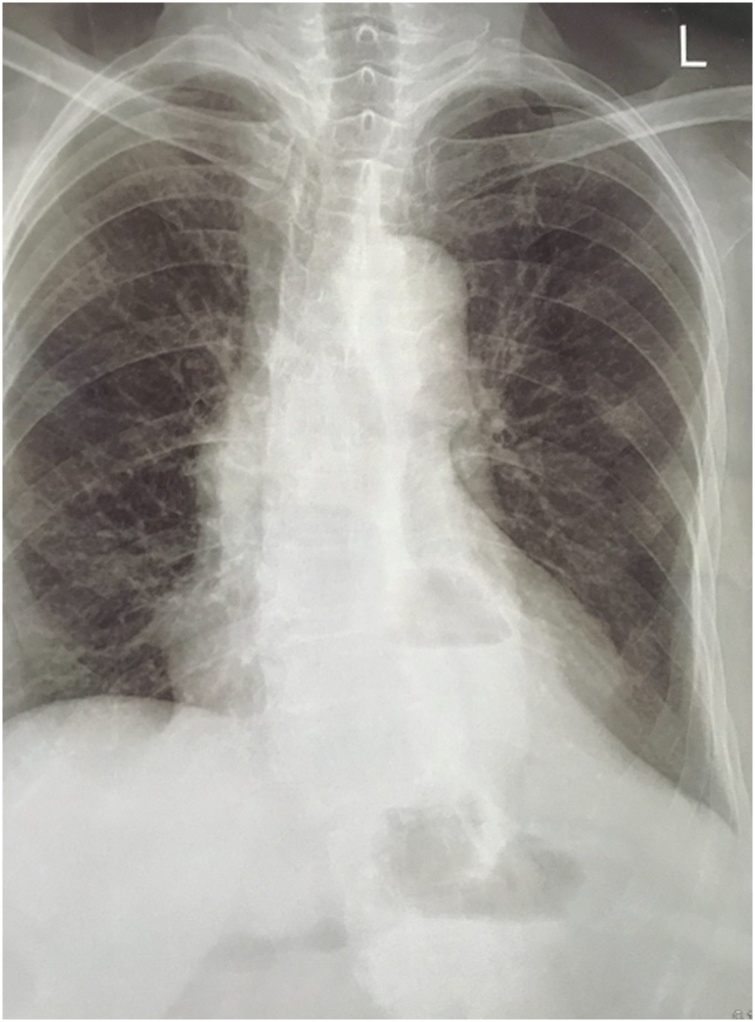
X-ray at six months follow up.

A 70-year-old woman presented with complaints of shortness of breath, abdominal pain and fullness. The patient had a history of laparoscopic diaphragmatic hernia surgery with the installation of an anti-adhesive mesh seven months ago. From chest X-ray and Computed Tomography (CT) scan, recurrence of diaphragmatic hernia was found. CT scan in Fig. 2 showed partial gastric outlet obstruction. The patient then received laparotomy, releasing the contents of the hernia in the form of transverse colon, ileum, gastric, and omentum. The defect was repaired using a self-attached mesh on the supra diaphragm (Fig. 3) through the abdominal approach (Fig. 4). The patient was hospitalised four days after surgery; no complaints were found at two weeks and six months follow-up. CT scan and chest X-ray (Fig. 5) were performed, and the results were normal.

Surgical procedures:

1 We performed surgical procedure under general anaesthesia.  
2 We made an upper midline incision.  
3 Next, we did a general exploration of the abdomen while paying attention to the diaphragmatic hernia in the area of the esophagogastric junction.
4 We mobilised the left hemi liver by dividing the triangular ligament to expose the diaphragmatic hernia.  
5 After that, we performed adhesiolysis and then divided and exposed the medial and lateral crus of the diaphragm.
6 Then, we released and downward-mobilised the gastric fundus, transverse colon, and ileum which enter the diaphragmatic hernia.
7 Afterwards, a self-attached mesh sized 14 × 9 cm was inserted into the superior of the diaphragm via diaphragmatic hernia with self-attached surface facing the abdomen without fixation suture.
8 Subsequently, we repaired the hernia with simple sutures to approximate the lateral and medial crus. The evaluation was done by using the index finger to feel whether it cannot enter the diaphragmatic hernia, and make sure it was not too tight.

## 3 Discussion

The incidence of non-traumatic diaphragmatic hernia in adults is rare with various clinical manifestations. Most cases can be associated with penetrating diaphragm trauma or blunt abdominal trauma [6,7,10].

In this case, the type of diaphragmatic hernia that occurred was type IV, with organ herniation containing transverse colon, ileum, gastric, and omentum. Management of a diaphragmatic hernia should be done by restoring the abdominal organ to its position and repairing the defect. The surgical approach for this pathology depends on the presence of visceral complications. There are two types of surgical approaches, thoracic and abdominal approaches. In an elective setting most authors recommend the thoracic approach, while, when there are septic complications, the abdominal approach is preferred. The current trend is to use minimally invasive surgical techniques such as laparoscopy, and especially thoracoscopy, which has been satisfactorily performed in adults. In the emergency setting, there is no consensus nor an absolute indication of the best timing of the surgery [7,8,13]. In this case, we used the abdominal approach in the emergency setting because of the partial obstruction resulting in the abdominal pain and fullness felt by the patient.

The use of surgical mesh in the management of a diaphragmatic hernia has developed and become a standard in recent years. Several types of mesh were developed. In general, diaphragmatic hernia repair uses anti-adhesive mesh. It is a second-generation multifilament mesh that is partially absorbable in approximately 20 days and has the benefit of anti-adhesion properties. There is one side of the mesh that prevents adhesion and the other side which is directed toward the abdominal wall or diaphragm to allow mesh ingrowth [8,9,13]. However, there is a study that showed anti-adhesive properties in the parietex mesh do not provide significant adhesion prevention [5]. In this case, we used a self-attached mesh, the Covidien ProGrip™ self-fixating mesh that is usually used in inguinal hernia repair cases. The patient experienced recurrence and adhesion to the diaphragmatic hernia area, therefore we used the ProGrip™ mesh and attached it to the superior side of the diaphragm through the gap between medial and lateral crus.

The purpose of this report is to present our experience in managing recurrence and adhesion of the diaphragmatic hernia with some technical modifications to minimise the complications and recurrences. We attached the hernia mesh onto the superior side of the diaphragm to reduce the risk of adhesion with the abdominal visceral organs. Postoperatively, the patient was hospitalised for four days. We followed up for six months, and no complaints were found. We also performed CT scan and chest X-ray, and the results were normal. However, further studies are still needed with a larger sample, and a more extended follow-up period.

## 4 Conclusions

The abdominal approach using supra-diaphragmatic self-attached mesh is a viable option in recurrent diaphragmatic hernia therapy. The six-month follow-up showed a safe procedure with no symptoms of recurrence and adhesion. In the future, we need to do a larger scale of research so that this approach can become a full technical report.

## Sources of funding

The authors declare that this study had no funding resource.

## Ethical approval

The informed consent form was declared that patient data or samples will be used for educational or research purposes. Our institutional review board also do not provide an ethical approval in the form of case report.

## Consent

We have obtained all patient’s consent and had the statement included in the consent section in the manuscript. We also do not include any of the patients name or the institution.

## Author contribution

Adeodatus Yuda Handaya conceived the study. Aditya Rifqi Fauzi and Victor Agastya Pramudya Werdana drafted the manuscript and critically revised the manuscript for important intellectual content. Adeodatus Yuda Handaya, Aditya Rifqi Fauzi, and Victor Agastya Pramudya Werdana facilitated all project-related tasks.

## Registration of research studies

There is in literature already published data stating Lapatoromy from human. This is not the first one.

## Guarantor

Adeodatus Yuda Handaya.

## Provenance and peer review

Not commissioned, externally peer-reviewed

## CRediT authorship contribution statement

**Adeodatus Yuda Handaya:** Conceptualization, Methodology, Resources, Writing - original draft. **Aditya Rifqi Fauzi:** Writing - review & editing, Resources, Validation. **Victor Agastya Pramudya Werdana:** .

## Declaration of Competing Interest

No potential conflict of interest relevant to this article was reported.
